# Supplementary material for: Slower EEG alpha generation, synchronization and “flow”—possible biomarkers of cognitive impairment and neuropathology of minor stroke
Source: PeerJ. 2017 Sep 28;5:e3839. doi: 10.7717/peerj.3839 (PMC5623310; doi:10.7717/peerj.3839)
Supplement: Supplemental Information 1 [file peerj-05-3839-s001.docx]

Stroke induced the transient cognitive impairment alongside the slowing of EEG alpha.

Stroke highly synchronized EEG alpha above the overall ipsi-lesional hemisphere.

Stroke highly synchronized EEG alpha inter-hemispherically above the frontal cortex.

Post-stroke frontal inter-hemispheric “alpha flow” presents a compensatory phenomenon.

EEG alpha slowing, alpha synchronization and “flow” are possible biomarkers of stroke.
