# Supplementary material for: Slower EEG alpha generation, synchronization and “flow”—possible biomarkers of cognitive impairment and neuropathology of minor stroke
Source: PeerJ. 2017 Sep 28;5:e3839. doi: 10.7717/peerj.3839 (PMC5623310; doi:10.7717/peerj.3839)
Supplement: Supplemental Information 3 — Bold numbers indicate statistically significant mean values at p ≤ 0.05. [file peerj-05-3839-s003.docx]

**Suppl Table 2 for Fig. 4. The mean intra-hemispheric coherence in the subacute and chronic stages of stroke patients vs. healthy controls.**

|  |  | **Delta** | **Theta** | **Alpha** | **Beta** | **Gamma** |
| --- | --- | --- | --- | --- | --- | --- |
| **F3 – F7** | *Control* | 0.38 ± 0.18 | 0.41 ± 0.18 | 0.53 ± 0.16 | 0.45 ± 0.17 | 0.40 ± 0.18 |
|  | *Subacute Stroke* | **0.44 ± 0.10** | **0.45 ± 0.12** | 0.56 ± 0.12 | **0.37 ± 0.14** | **0.28 ± 0.14** |
|  | *Chronic Stroke* | 0.36 ± 0.18 | 0.40 ± 0.20 | 0.51 ± 0.17 | **0.38 ± 0.19** | **0.28 ± 0.14** |
| **F3 – T5** | *Control* | 0.14 ± 0.04 | 0.14 ± 0.05 | 0.16 ± 0.06 | 0.14 ± 0.04 | 0.13 ± 0.05 |
|  | *Subacute Stroke* | **0.22 ± 0.08** | **0.21 ± 0.08** | **0.20 ± 0.07** | 0.15 ± 0.06 | 0.13 ± 0.05 |
|  | *Chronic Stroke* | 0.15 ± 0.05 | 0.14 ± 0.03 | 0.17 ± 0.04 | 0.13 ± 0.03 | 0.12 ± 0.03 |
| **F3 – P3** | *Control* | 0.14 ± 0.05 | 0.11 ± 0.02 | 0.17 ± 0.08 | 0.11 ± 0.02 | 0.12 ± 0.06 |
|  | *Subacute Stroke* | 0.14 ± 0.07 | **0.13 ± 0.05** | 0.17 ± 0.04 | 0.10 ± 0.01 | 0.10 ± 0.03 |
|  | *Chronic Stroke* | 0.13 ± 0.04 | 0.10 ± 0.02 | **0.19 ± 0.06** | 0.10 ± 0.01 | 0.10 ± 0.02 |
|  |  |  |  |  |  |  |
| **F4 – F8** | *Control* | 0.30 ± 0.12 | 0.28 ± 0.10 | 0.39 ± 0.11 | 0.36 ± 0.10 | 0.34 ± 0.15 |
|  | *Subacute Stroke* | 0.34 ± 0.15 | 0.27 ± 0.06 | **0.45 ± 0.08** | **0.31 ± 0.11** | **0.24 ± 0.12** |
|  | *Chronic Stroke* | **0.40 ± 0.14** | **0.37 ±0.11** | **0.51 ± 0.10** | 0.36 ± 0.10 | **0.26 ± 0.13** |
| **F4 – T6** | *Control* | 0.14 ± 0.05 | 0.11 ± 0.03 | 0.15 ± 0.06 | 0.11 ± 0.03 | 0.12 ± 0.06 |
|  | *Subacute Stroke* | 0.16 ± 0.08 | 0.12 ± 0.02 | **0.18 ± 0.07** | 0.12 ± 0.02 | 0.11 ± 0.03 |
|  | *Chronic Stroke* | 0.13 ± 0.04 | 0.11 ± 0.03 | **0.17 ± 0.05** | 0.11 ± 0.02 | 0.10 ± 0.01 |
| **F4 – P4** | *Control* | 0.18 ± 0.07 | 0.12 ± 0.04 | 0.19 ± 0.09 | 0.11 ± 0.03 | 0.15 ± 0.07 |
|  | *Subacute Stroke* | 0.18 ± 0.08 | **0.16 ± 0.04** | **0.22 ± 0.08** | 0.11 ± 0.02 | **0.11 ± 0.03** |
|  | *Chronic Stroke* | **0.13 ± 0.04** | 0.12 ± 0.04 | **0.22 ± 0.08** | 0.11 ± 0.02 | **0.10 ± 0.02** |

Bold numbers indicate statistically significant mean values at p ≤ 0.05.
